# Absolute and Relative Handgrip Strength as Indicators of Self-Reported Physical Function and Quality of Life in Breast Cancer Survivors: The EFICAN Study

**DOI:** 10.3390/cancers13215292

**Published:** 2021-10-21

**Authors:** Alba Esteban-Simón, David M. Díez-Fernández, Eva Artés-Rodríguez, Miguel Á. Casimiro-Artés, Manuel A. Rodríguez-Pérez, Herminia Moreno-Martos, Antonio J. Casimiro-Andújar, Alberto Soriano-Maldonado

**Affiliations:** 1 Department of Education, Faculty of Education Sciences, University of Almería, 04120 Almería, Spain; daviddiez@ual.es (D.M.D.-F.); manolo.rodriguez@ual.es (M.A.R.-P.); casimiro@ual.es (A.J.C.-A.); asoriano@ual.es (A.S.-M.); 2 CERNEP Research Centre, SPORT Research Group (CTS-1024), University of Almería, 04120 Almería, Spain; 3 Area of Statistics and Operative Research, Department of Mathematics, Faculty of Sciences, University of Almería, 04120 Almería, Spain; eartes@ual.es; 4 Realtrack Systems S. L., 04009 Almería, Spain; miguelcasi@correo.ugr.es; 5 Servicio Andaluz de Salud, Unidad de Gestión Clínica Almería Periferia, Distrito Sanitario Almería, 04009 Almería, Spain; herminiam.moreno.sspa@juntadeandalucia.es

**Keywords:** cancer, physical fitness, muscular strength, disability, life satisfaction, depression

## Abstract

**Simple Summary:**

Breast cancer is the most diagnosed type of cancer worldwide and it has a high survival rate. Thus, side effects related to breast cancer and treatments compromise lots of people’s physical functions and health-related quality of life. For this reason, it is important to manage these side effects in the follow up after treatments. The handgrip strength and the handgrip strength relative to body mass index may constitute useful, simple, quick and economically feasible tools that may help clinicians detecting these side effects, which is key to undertake actions for improving the physical function and health-related quality of life of breast cancer survivors.

**Abstract:**

Background: Although breast cancer (BC) is the most prevalent type of cancer in the world, its high survival rate implies that many people live long after the treatments and face their side effects. The physical function (PF) and health-related quality of life (HRQoL) of people surviving BC decreases significantly, which makes important to identify markers that may be associated with a better health status and prognosis. Previous studies suggest that handgrip strength (HGS) and HGS relative to the body mass index (rHGS) are good indicators of PF and HRQoL in different populations. However, it is unknown whether this applies to BC survivors. This study aimed to evaluate the association of HGS and rHGS with PF and HRQoL in this population. Methods: Sixty female BC survivors participated. Handgrip strength was assessed with a dynamometer. Arm volume was estimated and upper limb impairments, as well as cancer-related fatigue, depression, life satisfaction and HRQoL, were assessed using standardized questionnaires. Results: Higher levels of HGS and rHGS were associated with higher levels of HRQoL, lower cancer-related fatigue, and fewer problems with the affected arm. Conclusions: These results suggest that HGS may be a good indicator of self-reported PF and HRQoL in female BC survivors.

## 1. Introduction

Breast cancer has the highest incidence worldwide with more than 2.2 million new diagnoses in 2020 [1]. In Europe, there were 531,086 new diagnoses during 2020 [1]. According to the Spanish Association against Cancer (AECC) [2], 33,307 new cases were diagnosed during 2019. Nevertheless, ongoing advances in early detection and treatment of breast cancer have led to a significant mortality reduction. Breast cancer is the type of cancer with the highest survival rate following diagnosis, with 86% of patients surviving after 5-year [3]. Such a high survival rate leads to more people living with the side effects of the disease and its core treatments (chemotherapy, surgery, and radiotherapy) [4]. These side effects include the presence of lymphedema [5], cancer-related fatigue [6], shoulder–arm disabilities [7,8], lower levels of cardiorespiratory fitness [9,10] and muscular strength [11,12], sarcopenia [13], cardiac toxicity [14] or depression [15], among others. This implies a considerable decrease in patients’ health-related quality of life (HRQoL) [16], and could imply more difficulties to perform basic daily life tasks, such as dressing, combing hair, working, shopping, exercising, etc.

Muscular strength is a powerful marker of present and future health in children [17], adolescents [18,19], adults [20] and older adults [21]. In general population, higher levels of muscle strength have shown to be related to a lower risk of developing cardiovascular disease [22], type 2 diabetes [20], fragility [23] and mortality [24]. Specifically, handgrip strength (HGS), and HGS relative to the body mass index (BMI), have been proposed as highly relevant health indicators in different populations, such as young and older adults [25], adult males [26], aging adults [27], women with systemic lupus erythematosus [28], and women with fibromyalgia [29]. HGS and relative HGS (rHGS) have also been proposed as a cardiometabolic risk predictor in children and adolescents [30] and in middle-aged and older people [31,32], and as an all-cause [33,34,35] and cancer [36] mortality predictor, allowing to obtain useful information from a simple, quick, and very low-cost assessment.

The relationship of HGS with physical function and HRQoL in female breast cancer survivors has been studied. Higher HGS was associated with less treatment-related disability [37], less fear when using the affected limb in addition to less perceived weakness and less pain in the affected arm [38], greater activity of the upper limbs [38], reduced presence of depressive symptoms [39,40], higher self-reported HRQoL [41,42], less cancer-related fatigue [43,44] and a more favorable body composition [45]. However, information on the association of rHGS with health-related outcomes including HRQoL and cancer-associated side effects is scarce in female breast cancer survivors. RHGS is a good indicator of general health in different populations. For instance, higher levels of rHGS are associated with greater mobility in older adults [46,47], better cardiovascular health in obese women [48], lower prevalence of metabolic syndrome in adults [49], lower prevalence of osteoporosis in women [50], lower cardiometabolic risk in women with systemic lupus erythematosus [28], and lower risk of depressive mood in women [39]. Therefore, we speculated that higher rHGS would be related to better self-reported physical function and HRQoL in female breast cancer survivors.

Consequently, the aim of this study was to assess the association of HGS and rHGS with physical function and HRQoL in female breast cancer survivors. Additionally, the secondary aim was to explore if the association of HGS and rHGS of the affected and nonaffected arm with all the outcomes differed.

## 2. Materials and Methods

### 2.1. Design

A cross-sectional study was conducted with the baseline values of the EFICAN (Ejercicio FÍsico para pacientes con CÁNcer de mama) clinical trial [51].

### 2.2. Participants

Sixty women voluntarily participated in this study. They were recruited through different cancer-related associations and through announcements on social networks, press and radio in the city of Almería. The inclusion criteria were to be a breast cancer survivor and to have completed the core treatments of the disease (consisting of adjuvant chemotherapy, surgery and/or radiotherapy) in the 10 years prior to the beginning of the study. The exclusion criteria were the existence of pulmonary or cardiovascular disease, having metastatic breast cancer, planning a breast reconstruction intervention within 3 months of the beginning of the study and regularly performing >300 min per week of structured exercise. The study protocol was approved by the Ethics Committee of the Hospital Universitario Torrecárdenas de Almería, Spain (ref: Ejercicio-CáncerUAL [98/2019]). In addition, all the participants were duly informed and signed the informed consent form prior to being included in the study.

### 2.3. Protocol

Women interested in participating in the study filled out an online questionnaire asking for information about their disease and treatment. Participants who met the inclusion criteria underwent a medical evaluation to confirm that they met the study participation criteria. After obtaining medical authorization to participate in the study, the participants underwent a physical assessment at the University of Almería facilities. These assessments began by completing a series of questionnaires related to different quality of life variables. Then, the presence of lymphedema and handgrip strength were assessed.

### 2.4. Assessments

#### 2.4.1. Handgrip Strength

Handgrip strength was assessed using a digital dynamometer (TKK 5401 Grip-D, Takei Scientific Instruments Co. Ltd., Niigata, Japan), which has been used in previous studies on female breast cancer survivors [38,52,53,54]. Two attempts were made, alternating each hand, and the best result from each was selected. The mean value obtained was taken as the total score [55].

Handgrip strength relative to BMI was defined as HGS divided by BMI [46].

#### 2.4.2. Shoulder–Arm Disabilities

To assess shoulder–arm disabilities, we studied the arm volume (which is related to lymphedema) and the participants’ disability or difficulty in the upper limbs.

The arm volume was assessed by estimating the volume of each arm using the truncated cone formula [56]:

$$V=\frac{1}{12\pi }\sum_{i=1}^{n}L\left(C_{i}^{2}+C_{i}C_{i-1}+C_{i-1}²\right)$$

where *n* = number of segments, *L* = length of each segment, and *C_i_* and *C_i_*_−1_ = circumference at each end of the segment.

For this, the arm’s perimeter was measured at 6 different points (with 6 cm of separation between them) depending on the length of each participant’s arms following the protocol by Sander et al. [56] The difference of volume between arms were computed.

Upper-limb disability or difficulty was assessed using the Spanish version [57] of the Disabilities of the Arm, Shoulder, and Hand (DASH) questionnaire [37] with one optional additional module, intended to measure the extent of the injury to the upper limb when working. The final score ranged 0 to 100, where a higher number indicated greater disability or difficulties.

#### 2.4.3. Cancer-Related Fatigue

Cancer-related fatigue was measured using the Spanish version [58] of the Functional Assessment of Cancer Therapy-Fatigue (FACT-F) questionnaire [59]. The scores range from 0 to 52, where a higher score indicates lower fatigue.

#### 2.4.4. Depression

Depressive symptoms were assessed using the Spanish version [60] of the 20-item Center for Epidemiologic Studies-Depression Scale (CES-D) questionnaire [61]. The final score ranges from 0 to 60. A higher the total score means greater depressive symptoms.

#### 2.4.5. Life Satisfaction

Life satisfaction was assessed through the Satisfaction With Life Scale (SWLS) questionnaire [62], using its Spanish version [63], which has been validated for cancer patients [64]. The final score ranges from 0 to 25, where a greater score indicates greater satisfaction with life.

#### 2.4.6. Health-Related Quality of Life

HRQoL was assessed using the European Organization for Research and Treatment of Cancer, Quality of Life Questionnaire-Core30 (EORTC QLQ-C30) [65] in its Spanish version [66], with its complementary module for breast cancer European Organization for Research and Treatment of Breast Cancer, Cancer-Specific Quality-of-Life Questionnaire Module (EORTC QLQ-BR23) [67], also in its Spanish version [68]. Each of the subscale has a total score of 1–100, obtained as explained elsewhere [69], a higher score means a higher response level.

### 2.5. Statistical Analysis

Descriptive characteristics are presented in Table 1, using the mean and standard deviation for quantitative variables, and the number and frequency for categorical variables. The normality of the main variables was studied using histograms, Q-Q plots and through the Kolmogorov–Smirnov test. The association of HGS and rHGS with the aforementioned outcome variables was analyzed through partial correlation, adjusting for age, BMI and time since the disease diagnosis, to control for potential confounding. Finally, a linear regression analysis was performed including the variables related to physical function and quality of life as dependent variables in separate models, and the HGS as the independent variable, using the same adjustment variables. Homoscedasticity and linearity assumptions of the linear regression models, as well as the normality, no-multicollinearity and no-autocorrelation of the residuals were checked. In addition, the detection of possible outliers was studied through boxplots. Additional exploratory analyses were carried out to assess the potential differential association of the absolute and rHGS of the affected and non-affected with the above outcomes. All analyses were carried out using SPSS software (IBM SPSS Statistics for Windows, Version 27.0, IBM Corp., Armonk, New York, NY, USA). Statistical significance was set at *p* < 0.05.

## 3. Results

The descriptive characteristics of the study participants are presented in Table 1. Sixty female breast cancer survivors with an average age of 52.3 years (standard deviation [SD] 9.0) participated in this study. A total of two women were unable to complete the body composition assessments using bioimpedance because of heart problems (the presence of pacemakers) and feet problems. The average time since diagnosis was 4.5 (SD 3.1) years, with a maximum of 11 years since diagnosis, and the average HGS was 25.9 (SD 5.4) kg.

Table 2 shows the association between HGS and rHGS to arms volume difference and self-reported shoulder–arm disabilities, fatigue, depressive symptoms, life satisfaction and HRQoL, adjusting for the participants’ age, BMI and time since diagnosis. A negative association was found between HGS and rHGS to shoulder–arm disabilities (assessed using both the DASH questionnaire and the arm symptoms/discomfort subscale of the EORTC QLQ-BR23 module). Additionally, a positive association between HGS and rHGS to global health status/quality of life subscale of the EORTC QLQ-C30 questionnaire was found. Furthermore, a significant negative association was found between HGS and rHGS to fatigue (assessed using both FACIT-F questionnaire and the fatigue subscale of the EORTC QLQ-C30 questionnaire). There was no association of absolute or relative HGS with depressive symptoms, life satisfaction or any other quality of life subscale of the EORTC QLQ-C30 and EORTC QLQ-BR23 questionnaires (all *p* > 0.05).

Table 3 shows the results of the linear regression analysis assessing the association between HGS and rHGS to the variables that were previously significant in the partial correlation analysis, controlling for the potential confounding effects of age, BMI and time elapsed since diagnosis. As examples, an additional kg in the HGS test was associated with 0.653 (95% CI 0.262 to 1.044; ß = 0.397; *p* = 0.001) more units on the cancer-related fatigue scale, or with 1.114 (CI 95 % 0.115 to 2.113; ß = 0.289; *p* = 0.030) more units on the global health status/quality of life subscale of the EORTC QLQ-C30 questionnaire. Along the same lines, an additional kg of rHGS was associated with 12.589 (95% CI −19.914 to −5.265; ß = −0.445; *p* = 0.001) units less on the disabilities of the arm, shoulder and hand scale, with 7.911 (95% CI 4.176 to 11.645; ß = 0.515; *p* < 0.001) more units on the cancer-related fatigue scale, or with 14.650 (95% CI 5.296 to 24.003; ß = 0.408; *p* = 0.003) units on the global health status/quality of life subscale of the EORTC QLQ-C30 questionnaire.

The exploratory analyses assessing the specific associations of HGS and rHGS, differentially assessed with the affected and nonaffected arms, with all the outcomes are presented in Appendix A (Table A1). Overall, the association of the absolute and relative HGS of both arms showed a similar association with the outcomes.

## 4. Discussion

The main findings of the present study suggest that a higher absolute HGS is associated with fewer shoulder–arm disability, less cancer-related fatigue and greater HRQoL in female breast cancer survivors. Furthermore, a higher rHGS is significantly associated with less shoulder-arm disabilities, less cancer-related fatigue and higher HRQoL. These results suggest that both absolute and relative HGS may be good indicators of the health status of breast cancer survivors in the follow-up after core treatments. Particularly, the rHGS revealed relatively stronger associations with the studied outcomes.

No association of HGS, or rHGS with arms volume difference was observed. However, the present study observed a negative association of HGS and rHGS with shoulder–arm impairments. In this line, other studies [38,70], observed that higher HGS was associated with lower upper limb disability. Additionally, Lee et al. [38]. observed a negative association of HGS with subjective weakness of the affected arm, fear of using the affected arm, and recommendations from the medical professional to restrict the use of the affected arm and with limited activity in the affected arm. Future research should elucidate if upper limb impairments and limited physical activity might be associated to medical recommendations, which would highlight the great influence of medical support in the follow-up after completing breast cancer treatments, specifically, on shoulder–arm disability.

The negative associations observed between HGS and rHGS with cancer-related fatigue, support the findings obtained by Kalter et al. [43]., Kneis et al. [44]., and Cantarero-Villanueva et al. [52]. who observed that higher HGS was associated with lower cancer-related fatigue. Fatigue is one of the main side effects related to breast cancer treatments [6] and its significant increase during and after treatments cause a decrease in the patients’ quality of life, as well as in their ability to carry out daily life activities [71]. Then, considering the impact that fatigue may have on breast cancer survivors’ lives, assessing its presence becomes of major importance and absolute and relative HGS test may be very useful tools.

Despite what has been indicated in the previous literature [39,40,42,52], no association was observed between HGS, or rHGS, with depressive symptoms. This may be due to the use of different assessment instruments.

It has been shown that HRQoL is reduced after breast cancer core treatments [16]. The results presented in this study suggest that higher HGS and rHGS are associated with higher self-reported quality of life. These results are in line with those of Kaya et al. [41]. and Paek and Choi [42], who observed that lower HGS was associated with lower HRQoL, in spite of using different HRQoL scales. Further prospective research is needed to elucidate whether absolute or relative HGS might be associated with a lower decrease of HRQoL in the follow-up (e.g., 10 years as the context of this study) after completing the core treatments for breast cancer.

It should be noted that additional exploratory analyses were carried out to determine if HGS or rHGS of the affected and nonaffected arms could show stronger associations with the different self-reported physical function and quality of life studied outcomes, but the observed results were similar to previous analyses. Other studies analyzed the association of HGS of the affected arm [40,44,52] and HGS of the dominant hand [43,53] with physical function and HRQoL outcomes but any of them explored if using HGS of the affected and nonaffected arm or both could show stronger associations.

The handgrip strength test is a quick, simple and economically feasible assessment that may help clinicians initiate a process to identify the presence of shoulder–arm disabilities and cancer-related fatigue, as well as a decrease of quality of life in breast cancer survivors, which constitute three of the main side effects during and after cancer treatments. The detection of these side effects could lead to the implementation of actions for their improvement, causing a significant improvement on these patients’ well-being.

To the best of our knowledge, the present study is the first to examine the association of rHGS with different self-reported physical function and quality of life outcomes. According to these results, rHGS shows relatively stronger associations than HGS with all the analyzed outcomes. Consequently, rHGS could be speculated to be a better potential predictor of physical function and HRQoL.

This study also included standardized assessment instruments for this population, which has shown relevant associations that may contribute to the inclusion of HGS assessment in clinical practice, facilitating the early detection of breast cancer treatments side effects, and their improvement. However, further prospective research is needed to evaluate the utility of implementing the handgrip strength test in clinical practice to predict the deterioration of function and patient-reported outcomes following treatments.

This study has limitations that must be highlighted. The cross-sectional design precludes the establishment of causal relationships. The sample size was relatively low and studies with larger samples are needed to confirm or contrast these findings. Additionally, women included in the study had undergone breast cancer surgery and finished the core treatments up to 10 years before enrolment, which might result in a rather heterogeneous sample. Finally, we lacked information on the history of hormone therapy, which could affect muscle quality and strength.

## 5. Conclusions

In conclusion, the results of the present study suggest that both HGS and, particularly, rHGS could be good indicators of shoulder–arm disabilities, fatigue and decreased quality of life in breast cancer survivors.

## Figures and Tables

**Table 1:** Descriptive characteristics of the study participants.

Variable	*n*	Median	Mean	SD		Minimum	Maximum
Age (years)	60	53.2	52.3	9.0		28.8	70.2
Height (cm)	60	160.0	160.1	5.5		149.5	172.9
Weight (kg)	60	67.4	68.7	11.5		46.7	103.0
BMI (kg/m^2^)	60	26.4	26.9	4.7		18.7	40.0
Time since diagnosis (years)	60	4.0	4.5	3.1		0.0	11.0
HGS (kg)	60	25.9	25.9	5.4		13.1	34.2
rHGS (kg)	60	1.9	2.0	0.6		0.7	3.5
Arm volume difference ^a^ (%)	60	0.9	1.8	6.4		−12.3	29.1
DASH (range 0–100)	60	12.9	18.0	16.4		0.0	65.8
DASH work (range 0–100)	58	0.0	17.6	25.6		0.0	100.0
FACTI–F (range 0–52)	60	41.0	38.5	8.9		18.0	52.0
CES–D (range 0–60)	60	13.5	15.4	10.7		0.0	49.0
SWLS (range 0–25)	60	18.5	18.3	3.6		9.0	25.0
EORTC QLQ-C30							
QL2 subscale (range 0–100)	60	75.0	72.0	17.5		16.7	100.0
PF2 subscale (range 0–100)	60	93.3	86.9	14.3		33.3	100.0
RF2 subscale (range 0–100)	60	100.0	86.2	20.1		16.7	100.0
EF subscale (range 0–100)	60	75.0	75.0	23.8		0.0	100.0
CF subscale (range 0–100)	60	83.3	76.2	26.5		0.0	100.0
SF subscale (range 0–100)	60	83.3	71.5	33.3		0.0	100.0
FA subscale (range 0–100)	60	33.3	29.0	23.3		0.0	100.0
NV subscale (range 0–100)	60	0.0	0.6	3.0		0.0	16.7
PA subscale (range 0–100)	60	16.7	20.1	23.1		0.0	83.3
DY subscale (range 0–100)	60	0.0	12.4	18.5		0.0	66.7
SL subscale (range 0–100)	60	33.3	38.4	30.2		0.0	100.0
AP subscale (range 0–100)	60	0.0	4.0	10.9		0.0	33.3
CO subscale (range 0–100)	60	0.0	15.3	22.6		0.0	100.0
DI subscale (range 0–100)	60	0.0	2.8	9.4		0.0	33.3
FI subscale (range 0–100)	60	0.0	18.1	27.2		0.0	100.0
EORTC QLQ-BR23							
ST subscale (range 0–100)	60	21.4	21.8	17.0		0.0	71.4
AS subscale (range 0–100)	60	11.1	16.9	20.2		0.0	100.0
BS subscale (range 0–100)	60	8.3	18.1	19.4		0.0	100.0
BI subscale (range 0–100)	60	83.3	73.5	28.9		0.0	100.0
FU subscale (range 0–100)	60	66.7	52.8	32.6		0.0	100.0
SEF subscale (range 0–100)	60	33.3	28.3	20.4		0.0	83.3
**Variable**	***n***				**%**		
Affected arm							
Left	33				55.0		
Right	26				43.3		
Both	1				1.7		
Tumor type (*n* = 60)							
HR+/HER2−	39				65.1		
HR+/HER2+	11				18.3		
HR−/HER2+	2				3.3		
HR−/HER2−	8				13.3		
Treatment (*n* = 60)							
Chemotherapy	46				76.7		
Radiotherapy	52				86.7		
Surgical procedure (*n* = 60)							
Tumerectomy	41				68.3		
Mastectomy	18				30.0		
No surgery	1				1.7		
Lymph node resection (*n* = 60)	25				41.7		
Endocrine therapy (*n* = 60)	52				86.7		
Tamoxifeno	34				56.7		
Anastrozol	11				18.3		
Paclitaxel	7				11.7		
Trastuzumab	6				10.0		
Goserelina	5				8.3		
Letrozol	3				5.0		
Exemestano	1				1.7		

SD, standard deviation; HGS, handgrip strength; rHGS, relative handgrip strength; DASH, Disabilities of the Arm, Shoulder and Hand questionnaire; FACIT-F, Functional Assessment of Chronic Illness Therapy-Fatigue; CES-D, Center for Epidemiologic Studies Depression Scale; SWLS, Satisfaction with life scale; EORTC QLQ-C30, European Organization for Research and Treatment of Cancer; QL2, global health status/quality of life; PF2, physical function; RF2, task performance; EF, emotional performance; CF, cognitive performance; SF, social performance; FA, fatigue; NV, nausea and vomiting; PA, pain; DY, dyspnea; SL, insomnia; AP, loss of appetite; CO, constipation; DI, diarrhea; FI, financial difficulties; EORTC QLQ-BR23, European Organization for Research and Treatment of Cancer—breast cancer module; ST, side effects of therapy; AS, arm symptoms/discomfort; BS, breast symptoms/discomfort; BI, body image; FU, future perspective; SEF, sexual performance; RH, hormone receptor; HER2, HER2 protein; BSGC, selective sentinel lymph node biopsy. ^a^ Difference between the volume of the right and left arm (expressed in %).

**Table 2:** Partial correlation assessing the association of HGS, and rHGS with arms volume difference, shoulder–arm disabilities, fatigue, depressive symptoms, life satisfaction and self-reported HRQoL in female breast cancer survivors.

		HGS		rHGS	
Variable	*n*	r	*p*	r	*p*
Arms volume difference ^a^	60	−0.103	0.447	−0.073	0.589
DASH	60	−0.210	0.123	−0.418	0.001
DASH work module	58	−0.284	0.035	−0.361	0.006
FACIT-F	60	0.401	0.002	0.493	<0.001
CES-D	60	−0.122	0.376	−0.186	0.161
SWLS	60	0.069	0.617	0.099	0.459
EORTC QLQ-C30					
QL2 subscale	60	0.289	0.030	0.387	0.003
PF2 subscale	60	0.103	0.448	0.254	0.054
RF2 subscale	60	0.111	0.410	0.182	0.172
EF subscale	60	0.211	0.115	0.179	0.179
CF subscale	60	0.148	0.270	0.165	0.216
SF subscale	60	−0.093	0.491	0.188	0.157
FA subscale	60	−0.164	0.224	−0.274	0.038
NV subscale	60	−0.041	0.764	0.121	0.365
PA subscale	60	−0.152	0.258	−0.165	0.216
DY subscale	60	−0.005	0.972	−0.052	0.698
SL subscale	60	−0.030	0.826	−0.076	0.572
AP subscale	60	−0.246	0.065	−0.100	0.457
CO subscale	60	−0.100	0.458	0.023	0.862
DI subscale	60	0.014	0.920	0.067	0.619
FI subscale	60	0.167	0.215	−0.085	0.524
EORTC QLQ-BR23					
ST subscale	60	−0.139	0.304	−0.140	0.294
AS subscale	60	−0.346	0.008	−0.332	0.011
BS subscale	60	−0.025	0.856	−0.108	0.421
BI subscale	60	0.089	0.510	0.238	0.072
FU subscale	60	0.153	0.256	0.120	0.370
SEF subscale	60	0.078	0.565	0.197	0.138

r, partial correlation coefficient; *p*, *p*-value; DASH, Disabilities of the Arm, Shoulder and Hand questionnaire; FACIT-F, Functional Assessment of Chronic Illness Therapy-Fatigue; CES-D, Center for Epidemiologic Studies Depression Scale; SWLS, Satisfaction with life scale; EORTC QLQ-C30, European Organization for Research and Treatment of Cancer; QL2, global health status/quality of life; PF2, physical function; RF2, task performance; EF, emotional performance; CF, cognitive performance; SF, social performance; FA, fatigue; NV, nausea and vomiting; PA, pain; DY, dyspnea; SL, insomnia; AP, loss of appetite; CO, constipation; DI, diarrhea; FI, financial difficulties; EORTC QLQ-BR23, European Organization for Research and Treatment of Cancer—breast cancer module; ST, side effects of therapy; AS, arm symptoms/discomfort; BS, breast symptoms/discomfort; BI, body image; FU, future perspective; SEF, sexual performance. The analyses were adjusted for age, BMI, and time since diagnosis. ^a^ Difference between the volume of the right and left arm (expressed in %).

**Table 3:** Linear regression analysis assessing the association between HGS and rHGS to shoulder–arm disabilities, fatigue and HRQoL, controlling for the potential confounding effects of age, BMI and time elapsed since diagnosis.

	HGS					rHGS				
Variable	β	B	SD	95% CI	*p*	β	B	SD	95% CI	*p*
Weight	−0.040	−0.085	0.290	−0.666, 0.496	0.771	−0.591	−11.722	2.301	−16.331, −7.112	<0.001
DASH	−0.244	−0.740	0.382	−1.507, 0.026	0.058	−0.445	−12.589	3.656	−19.914, −5.265	0.001
DASH work module	−0.278	−1.311	0.607	−2.529, −0.093	0.035	−0.374	−16.349	5.750	−27.878, −4.821	0.006
FACIT-F	0.397	0.653	0.195	0.262, 1.044	0.001	0.515	7.911	1.864	4.176, 11.645	<0.001
EORC QLQ-C30										
QL2 subscale	0.289	1.114	0.499	0.115, 2.113	0.030	0.408	14.650	4.669	5.296, 24.003	0.003
FA subscale	−0.225	−0.967	0.552	−2.074, 0.140	0.085	−0.382	−15.407	5.243	−25.913, −4.900	0.005
EORTC QLQ-BR23										
AS subscale	−0.347	−1.300	0.476	−2.253, −0.346	0.008	−0.343	−11.993	4.546	−21.100, −2.886	0.011

β, standardized regression coefficient; B, non-standardized regression coefficient; SD, standard deviation; CI, confidence interval; *p*, *p*-value; BMI, body mass index; HGS, hand grip strength; DASH, Disabilities of the Arm, Shoulder and Hand questionnaire; FACIT-F, Functional Assessment of Chronic Illness Therapy-Fatigue; EORTC QLQ-C30, European Organization for Research and Treatment of Cancer; QL2, global health status/quality of life; EORTC QLQ-BR23, European Organization for Research and Treatment of Cancer—breast cancer module; AS, arm symptoms/discomfort. The analyses were adjusted for age, BMI (body mass index) and time since diagnosis, except for the body composition variables, where BMI was excluded as the adjustment variable.

## Data Availability

The data presented in this study are available on reasonable request and for research purposes from the corresponding author.

## Appendix A

**Table A1:** Partial correlation assessing the association of HGS, and rHGS of the affected and nonaffected arms with arms volume difference, shoulder–arm disabilities, fatigue, depressive symptoms, life satisfaction and self-reported HRQoL in female survivors of breast cancer.

Variable		Affected Arm				Nonaffected Arm			
		HGS		rHGS		HGS		rHGS	
	*n*	r	*p*	r	*p*	r	*p*	r	*p*
Arms volume difference ^a^	60	−0.083	0.554	−0.069	0.621	−0.107	0.444	−0.099	0.477
DASH	60	−0.255	0.066	−0.437	0.001	−0.127	0.363	−0.339	0.012
DASH work module	58	−0.346	0.011	−0.433	0.001	−0.211	0.129	−0.331	0.015
FACIT-F	60	0.373	0.006	0.490	<0.001	0.433	0.001	0.527	<0.001
CES-D	60	−0.069	0.622	−0.135	0.329	−0.137	0.330	−0.198	0.152
SWLS	60	−0.040	0.775	0.023	0.870	0.142	0.310	0.158	0.253
EORTC QLQ-C30									
QL2 subscale	60	0.188	0.177	0.319	0.019	0.267	0.053	0.387	0.004
PF2 subscale	60	0.085	0.546	0.332	0.014	0.076	0.590	0.320	0.018
RF2 subscale	60	0.132	0.347	0.306	0.024	−0.020	0.889	0.196	0.156
EF subscale	60	0.228	0.101	0.220	0.110	0.247	0.075	0.236	0.086
CF subscale	60	0.151	0.282	0.193	0.163	0.119	0.398	0.185	0.181
SF subscale	60	−0.134	0.338	0.215	0.119	−0.105	0.454	0.236	0.085
FA subscale	60	−0.131	0.352	−0.332	0.014	−0.150	0.284	−0.341	0.011
NV subscale	60	−0.169	0.227	−0.017	0.902	−0.186	0.181	−0.115	0.406
PA subscale	60	−0.105	0.454	−0.220	0.109	−0.039	0.780	−0.176	0.202
DY subscale	60	0.010	0.943	−0.108	0.437	0.070	0.616	−0.064	0.646
SL subscale	60	−0.041	0.768	−0.112	0.422	0.031	0.826	−0.077	0.582
AP subscale	60	−0.360	0.008	−0.311	0.022	−0.157	0.262	−0.171	0.216
CO subscale	60	−0.045	0.747	0.040	0.772	−0.073	0.604	0.022	0.876
DI subscale	60	−0.018	0.897	0.008	0.954	−0.123	0.381	−0.079	0.569
FI subscale	60	0.115	0.411	−0.157	0.256	0.171	0.222	−0.124	0.372
EORTC QLQ-BR23									
ST subscale	60	−0.159	0.255	−0.181	0.191	−0.076	0.591	−0.133	0.337
AS subscale	60	−0.344	0.012	−0.339	0.012	−0.229	0.099	−0.249	0.070
BS subscale	60	−0.002	0.990	−0.097	0.487	0.039	0.779	−0.060	0.664
BI subscale	60	0.069	0.625	0.241	0.079	0.193	0.167	0.326	0.016
FU subscale	60	0.207	0.137	0.139	0.316	0.045	0.750	0.024	0.864
SEF subscale	60	0.012	0.934	0.162	0.242	0.095	0.497	0.208	0.130

r, correlation coefficient; *p*, *p*-value; DASH, Disabilities of the Arm, Shoulder and Hand questionnaire; FACIT-F, Functional Assessment of Chronic Illness Therapy-Fatigue; CES-D, Center for Epidemiologic Studies Depression Scale; SWLS, Satisfaction with life scale; EORTC QLQ-C30, European Organization for Research and Treatment of Cancer; QL2, global health status/quality of life; PF2, physical function; RF2, task performance; EF, emotional performance; CF, cognitive performance; SF, social performance; FA, fatigue; NV, nausea and vomiting; PA, pain; DY, dyspnea; SL, insomnia; AP, loss of appetite; CO, constipation; DI, diarrhea; FI, financial difficulties; EORTC QLQ-BR23, European Organization for Research and Treatment of Cancer—breast cancer module; ST, side effects of therapy; AS, arm symptoms/discomfort; BS, breast symptoms/discomfort; BI, body image; FU, future perspective; SEF, sexual performance. The analyses were adjusted for age, BMI, and time since diagnosis. ^a^ Difference between the volume of the right and left arm (expressed in %).
